# Supplementary material for: Round-window delivery of lithium chloride regenerates cochlear synapses damaged by noise-induced excitotoxic trauma via inhibition of the NMDA receptor in the rat
Source: PLoS One. 2023 May 22;18(5):e0284626. doi: 10.1371/journal.pone.0284626 (PMC10202264; doi:10.1371/journal.pone.0284626)
Supplement: S1 Fig — A-C Original uncropped and unadjusted images representing for the β-actin (A), total NR2B (B), and phopho-NR2B (C). d = day, R = replicate. (DOCX) [file pone.0284626.s001.docx]

**S1 Fig. Western blot analysis of total NR2B, phospho-NR2B (pNR2B), β-actin protein expression levels in the cochlea.**

**
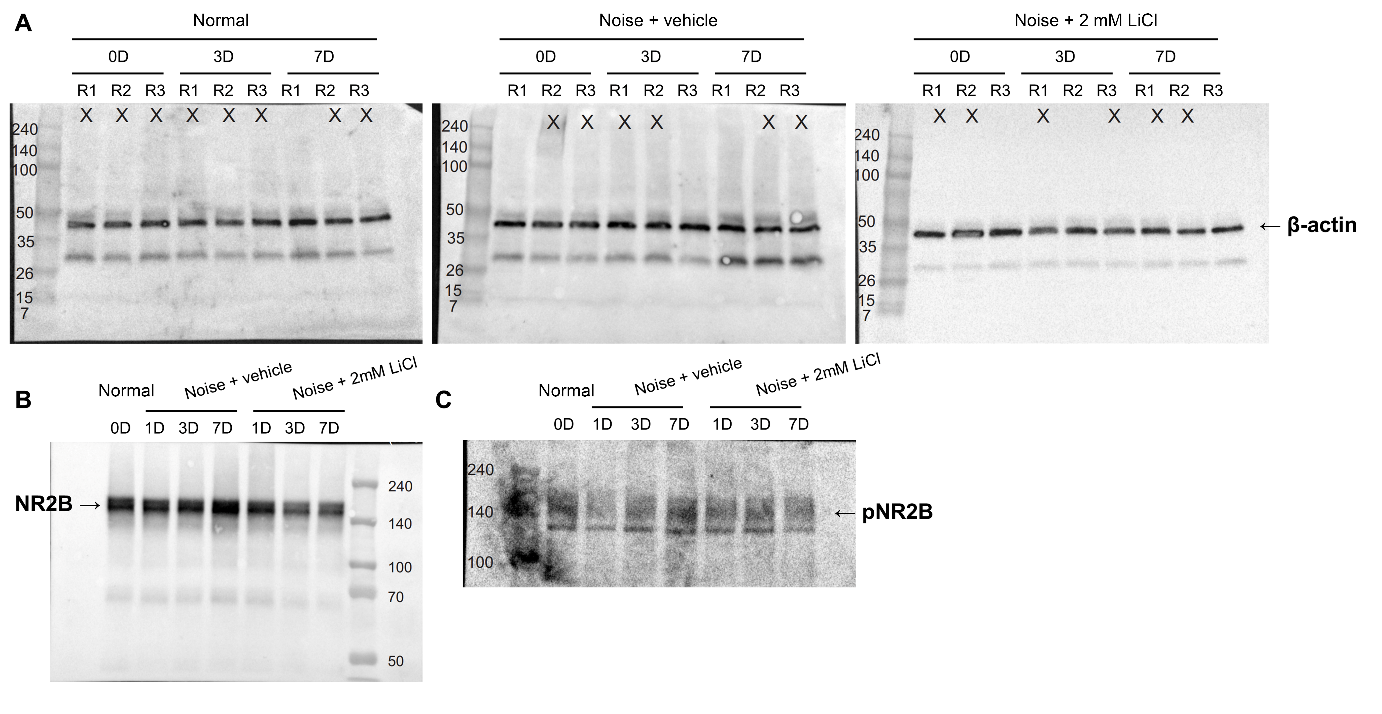
**

**A-C** Original uncropped and unadjusted images representing for the β-actin (A), total NR2B (B), and phopho-NR2B (C). d = day, R = replicate
