# Supplementary material for: Round-window delivery of lithium chloride regenerates cochlear synapses damaged by noise-induced excitotoxic trauma via inhibition of the NMDA receptor in the rat
Source: PLoS One. 2023 May 22;18(5):e0284626. doi: 10.1371/journal.pone.0284626 (PMC10202264; doi:10.1371/journal.pone.0284626)
Supplement: Attachment — Submitted filename: review comments.docx [file pone.0284626.s002.docx]

This manuscript investigated the effects of round-window delivery of lithium on noise-induced cochlear synaptopathy and hearing loss in a rat model. They also investigated the changes in NMDA receptor expression as potential underlying mechanisms. The experimental design is straightforward, although including a control + vehicle group would add more confidence for some of the conclusions (see specific comments below). The methods were appropriate and well described except the statistical analysis (see specific comments below). The results section could be improved with a more appropriate statistical analysis.

Since there is no page numbers and line numbers for the downloaded manuscript, the page numbers mentioned in the comments below is referred to the page number of the entire PDF file , which does not corresponding to the actual page numbers of the manuscript.

1. Title: The animal species needs to be included in the title
2. Page 9, Abstract:
   a. Please provide the dose (or dose range) of lithium chloride and clarify whether it was a single treatment or multiple treatment
   b. Throughout the whole abstract, there is no mentioning of what animals was used.
3. Page 10: “it could contribute to difficulties understanding …” should be “…difficulties in understanding…”.
4. Page 14, first line: Will the ABR testing at 3 days after treatment not only assess the “effects of the surgical manipulations”, but also the treatment effects?
5. Page 14, line 6: n = 2 for groups 1 and 2?
6. Page 15, line 1: What does “imaged at frequencies of 8, 12, 16 and 32 kHz” mean? Please clarify how the frequency location was determined.
7. Page 15, Western blot analysis: The statement “To assess NR2B activity” is not appropriate, as Western blot can only look at the protein expression of the receptor, but not the activity of the receptor.
8. Page 16, line 15: “followed by autoradiography”. “Autoradiography” is referred to the photochemical technique used to record the spatial distribution of radiolabeled compounds within a specimen or an object, which shouldn’t be used to describe the imaging analysis procedure for Western blot using ChemiDoc.
9. Pages 16 – 17: “To compare the ABR thresholds, synapse counts, and western blot result between experimental groups.” This is not a complete sentence.
10. Page 17: The sentence “parametric data were analyzed using the repeated measures one-way analysis of variance (ANOVA) was used with the Greenhouse-Geisser correction” is not grammatically correct.
11. Statistical analysis:
    a. For the ABR data, you have 5 groups (control, noise-only, noise + vehicle, noise + 1mM and noise + 2mM), 4 time pints (baseline, 3 days, 1 week and 2 weeks) and 4 frequencies (8, 12, 16 and 32). A 3-way ANOVA (group x time x frequency) with repeated measures or a linear mixed model analysis should be used. If you want to analyse each frequency separately, you should use a two-way ANOVA (group x time) with repeated measures or a linear mixed model analysis. An one-way ANOVA is not appropriate for this data. In addition, why the baseline data was not included in the analysis?
    b. For the wave I amplitude data (i.e., figure 3), no statistical method was mentioned. Assume that a one-way ANOVA was used, but looking at the data, at least a two-way ANOVA (time x stimulus level) with repeated measures should be used for each group instead, but a 3- way analysis would be even better.
12. Page 17, Results: The statement of “a temporary ~15 dB SPL threshold shift and a permanent ~15 dB SPL threshold shift” is not appropriate, as “temporary” means lasting for only a limited period of time; not permanent, but the threshold shift was “permanent” in your case. So, please rewrite this sentence.
13. Figure 2: The baseline ABR for all the groups should be included in order to see changes before and after noise exposure/drug treatment.
14. Page 17, Results: “The surgery for the round-window delivery of vehicle caused temporary increases in the 8 kHz and 12 kHz thresholds for only one week after the surgery, as observed by comparing the noise-only and noise + vehicle groups”. Without a control + vehicle group, the conclusion that “the surgery for the round-window delivery of vehicle caused temporary increases in the 8 kHz and 12 kHz thresholds” cannot be made. As shown in Fig 2, there seems no difference in the threshold between 3 days and 1 week for the noise + vehicle group (there seems slight improvement).
15. Page 18, Results: “All experimental groups had statistically significant differences before and after noise exposure as determined by one-way ANOVA with the Greenhouse-Geisser correction for all levels from 65 -80 dB SPL”. This statement is not backed up by the one-way ANOVA analysis, as you were talking about two factors here, i.e., time and levels. It is not clear how the one-way ANOVA could be run to tell you the differences for the two factors. As commented on the statistical analysis, a two-way ANOVA or 3-way ANOVA/linear mixed model should be used.
16. Figure 3 legend: what does the grey area mean? The exposed intensity? Please clearify.
17. Page 19, Results: For the synapses data, it is also better to do a 2-way ANOVA or linear mixed model analysis for similar reasons mentioned above. In addition, n = 2 seems a very small sample size for valid statistical analysis.
18. Figure 5: please add sample size for each group. From the western blot image, there seems only one sample in the control group and 2 samples in the other two groups. Please clarify.
19. The Supplementary figure seems not necessary.
