# Supplementary material for: Round-window delivery of lithium chloride regenerates cochlear synapses damaged by noise-induced excitotoxic trauma via inhibition of the NMDA receptor in the rat
Source: PLoS One. 2023 May 22;18(5):e0284626. doi: 10.1371/journal.pone.0284626 (PMC10202264; doi:10.1371/journal.pone.0284626)
Supplement: Attachment — Submitted filename: rebuttal letter_R2.docx [file pone.0284626.s004.docx]

**Rebuttal Letter**

**Manuscript ID:** PONE-D-22-25555R1

**Title of Manuscript**: Round-window delivery of lithium chloride protects cochlear synapses from noise-induced excitotoxic trauma by inhibiting NMDA receptor

*Authors’ Comment*

*We would like to thank the associate editor and the two reviewers for their valuable time and constructive comments. Below please find our response to the reviewers’ comments. Their comments are listed below followed by our responses summarizing how we addressed their concerns. We also included page numbers to indicate where changes were made in the manuscript. We hope that we have satisfactorily addressed the reviewers’ concerns. Due to insightful comments provided by the reviewers, the quality of our manuscript has been substantially improved. We want to thank the two reviewers and the editors for their valuable time and effort.*

Reviewer #1: Thank you for the changes you made to the manuscript.

I have a couple of minor revisions that I think need to be addressed prior to publication.

Suggested change to the title from "Round-window delivery of lithium chloride regenerates rat cochlear synapses from noise-induced excitotoxic trauma by inhibiting NMDA receptor" to one of the following:

"Round-window delivery of lithium chloride regenerates cochlear synapses damaged by noise-induced excitotoxic trauma via inhibition of the NMDA receptor in the rat"

OR

"Regeneration of cochlear synapses in the rat following noise-induced excitotoxic trauma by round-window delivery of lithium chloride to inhibit the NMDA receptor" or something similar, because I don't think "regenerates rat cochlear synapses from noise-induced excitotoxic trauma" makes sense.

***Author’s Response: Thank you for your comment. We changed the title to "Round-window delivery of lithium chloride regenerates cochlear synapses damaged by noise-induced excitotoxic trauma via inhibition of the NMDA receptor in the rat".***

Line 208: "the two-way repeated measures one-way analysis of variance" Was it one way or two way?

***Author’s Response: Thank you for your careful observation. There was typo. We used a two-way repeated measures analysis of variance (ANOVA).***

Line 224 "in the noise + vehicle group had returned to that in the noise-only group." change to ...of the noise-only group.

***Author’s Response: Thank you for your careful observation. We changed to “of”.***

Line 302 "There is a robust literature on potential therapeutic agent to prevent or regenerate noise-induced synaptic loss in the cochlea [7, 23-26]. " Change to "There is a robust literature on the delivery of potential therapeutic agents to prevent or regenerate noise-induced synaptic loss in the cochlea [7, 23-26].

***Author’s Response: Thank you for your comment. We changed the sentence.***

Suggested change to Line 371 ""Fourthly, many key questions remain for clinical application. There is likely a significant clinical population with loss of cochlea synapse that could be addressed by a treatment analogous to that used here in the noise-exposed rat. Although the local delivery of lithium to the round window was effective in regenerating cochlear synapses 24 hours after the noise exposure, a key question would be how long this time window could be extended for effective synaptogenesis in a clinical setting."

***Author’s Response: Thank you for your comment. We changed the last sentences as reviewer’s comment.***

Reviewer #2: The authors have addressed most of my comments to my satisfaction. However, for the ABR results, although the authors added the control group to Figure 2, the control group was still excluded from the statistical analysis. Without statistically analyse the difference between control and other experimental groups, especially, between control and noise + drug groups, a piece of very important information is missing. That is whether or not the drug treatment would have any therapeutic value/potential, i.e., to make the hearing threshold return to the control level. Therefore, control group should be included in the analysis and the results need to be discussed accordingly. In addition, in lines 208 – 208, please clarify what does “the two-way repeated measures one-way analysis of variance (ANOVA)” mean?

***Author’s Response: We fully understand the reviewer’s concerns. The purpose of this study was to compare the protective effects on cochlea synapses after noise exposure. To achieve this, the control group should have similar variables to the experimental group except for not receiving lithium administration. Therefore, controls included animals exposed to noise who received only the vehicle (see abstract line 29-30). To avoid confusion, we have changed the term ‘control group’ to ‘normal group’ in the manuscript and figures. Additionally, we have corrected a typographical error in the statistical description in line 208 as follows: the two-way repeated measures analysis of variance (ANOVA).***
